# Availability of emergency obstetric and newborn care services at public health facilities of Sindh province in Pakistan

**DOI:** 10.1186/s12913-019-4830-6

**Published:** 2019-12-16

**Authors:** Ramesh Kumar, Jamil Ahmed, Fozia Anwar, Ratana Somrongthong

**Affiliations:** 1 Health Services Academy, Islamabad, Pakistan; 2 Department of Family and Community Medicine, College of Medicine and Medical Sciences, Arabian Gulf University, Manama, Bahrain; 3 COMSATS Institute of Information Technology, Islamabad, Pakistan; 4 College of Public Health Sciences, Chulalongkorn University, Bangkok, Thailand

**Keywords:** Maternal health, Newborn care, Basic and comprehensive emergency obstetric and newborn care

## Abstract

**Background:**

Basic and comprehensive emergency obstetric care services in Pakistan remain a challenge considering continued high burden of maternal and newborn mortality. This study aimed to assess the availability of emergency obstetric and newborn care in Sindh Province of Pakistan.

**Methods:**

This cross-sectional survey was conducted in twelve districts of the Sindh province in Pakistan. The districts were selected based on the maternal neonatal and child health indicators. Data were collected from 63 public-sector health facilities including district, *Taluka* (subdistrict) headquarters hospitals and rural health centers. Basic and comprehensive emergency obstetric newborn care services were assessed through direct observations and interviews with the heads of the health facilities by using a World Health Organization pretested and validated data collection tool. Participants interviewed in this study included the managers and auxiliary staff and in health facilities.

**Results:**

Availability of caesarean section (23, 95% C.I. 14.0–35.0) and blood transfusion services (57, 95% CI. 44.0–68.0), the two components of comprehensive emergency obstetric and newborn care, was poor in our study. However, assessment of the seven components of basic emergency obstetric and newborn services showed that 92% of the health facilities (95% C.I. 88.0–96.0) had parenteral antibiotics, 90%, (95% C.I. 80.0–95.0) had oxytocin, 92% (95% CI 88.0–96.0) had manual removal of the placenta service, 87% (95%, C.I. 76.0–93.0) of the facilities had staff who could remove retained products of conception, 82% (95% C.I. 71.0–89.0) had facilities for normal birth and 80% (95% C.I. 69.0–88.0) reported presence of neonatal resuscitation service.

**Conclusion:**

Though the basic obstetric and newborn services were reasonably available, comprehensive obstetric and newborn services were not available as per the World Health Organization’s standards in the surveyed public health facilities. Ensuring the availability of caesarean section and blood transfusion services within these facilities may improve population’s access to these essential services around birth.

## Background

In 2015, 303,000 maternal deaths were reported globally, of which 99% occurred in developing countries. About 60% of this global smaternal mortality burden is shared by only ten countries including India, Nigeria, Afghanistan, Ethiopia, Democratic Republic of the Congo, Tanzania, Kenya, Uganda, Bangladesh, and Pakistan [1]. A lack of access and availability of emergency obstetric care accounts for a large majority of maternal mortality in these countries [2]. Although maternal mortality ratio in Pakistan declined from 521 in 1990 to 178 in 2015, the country still faces a high maternal mortality ratio compared to the regional countries. In bordering India, maternal mortality ratio declined from 556 to 174, in Bangladesh from 569 to 176, in Afghanistan from 1340 to 396, between 1990 and 2015 [3]. The Pakistan Demographic and Health Survey in 2012–2013 reported a maternal mortality ratio of 276 per 100, 000 and a neonatal mortality rate of 55 per 1000 [4]. In the province of Sindh, the maternal mortality ratio reported was 314, which was much higher than the national average, indicating wide disparities within country with regard to access to maternal and child healthcare services [5]. The country also faltered in its achievement of Millennium Development Goal of reducing maternal mortality ratio by three quarters, between 1990 and 2015 [6]. Such a failure is reflected by the fact that maternal mortality burden has increased from previous estimates for some parts of Pakistan. For instance, the maternal mortality ratio in Thatta district, in Southern Sindh province, increased more than 50% from 219 in 2010 to 333 per 100,000 live births in 2013 [7].

Pakistan’s maternal and child health burden continues to remain high and coverage of maternal survival interventions is still low despite the investments in recent years, by both public sector and non-governmental organizations. The country’s total maternal, newborn and child health expenditure increased by 67% between 2001 and 2010 [8]. Maternal and newborn deaths in Pakistan could be prevented by improving the access to and availability of basic emergency obstetric and newborn care (BEmONC) and comprehensive emergency obstetric and newborn care (CEmONC) [9].

BEmONC and CEmONC services are offered at various tiers of public healthcare system. BEmONC services including the normal vaginal deliveries, administer oxytocin, newborns resuscitation services are expected to be provided at primary healthcare facilities. CEmONC services were offered for breech presentation, prolonged labor and caesarean section, blood transfusion and care of sick newborns is provided at secondary and tertiary care hospitals. In Sindh, the health system consists of basic health units (BHUs), rural health centers (RHCs), *Taluka* (subdistrict) headquarter hospitals (THQs), mother and child health (MCH) centers and district headquarters hospitals (DHQs) or civil hospitals. Despite of health facility availability in almost all administrative areas in the province, the availability of and access to quality maternal and newborn care services has been poor [10, 11]. The present study was sanctioned considering the need for an evaluation of the essential services and resources related to maternal and newborn care provision in the province of Sindh. The study was aimed to encompass the level of infrastructure, equipment and commodities required to deliver emergency obstetric and newborn care services in the province.

## Methods

**Fig. 1:**
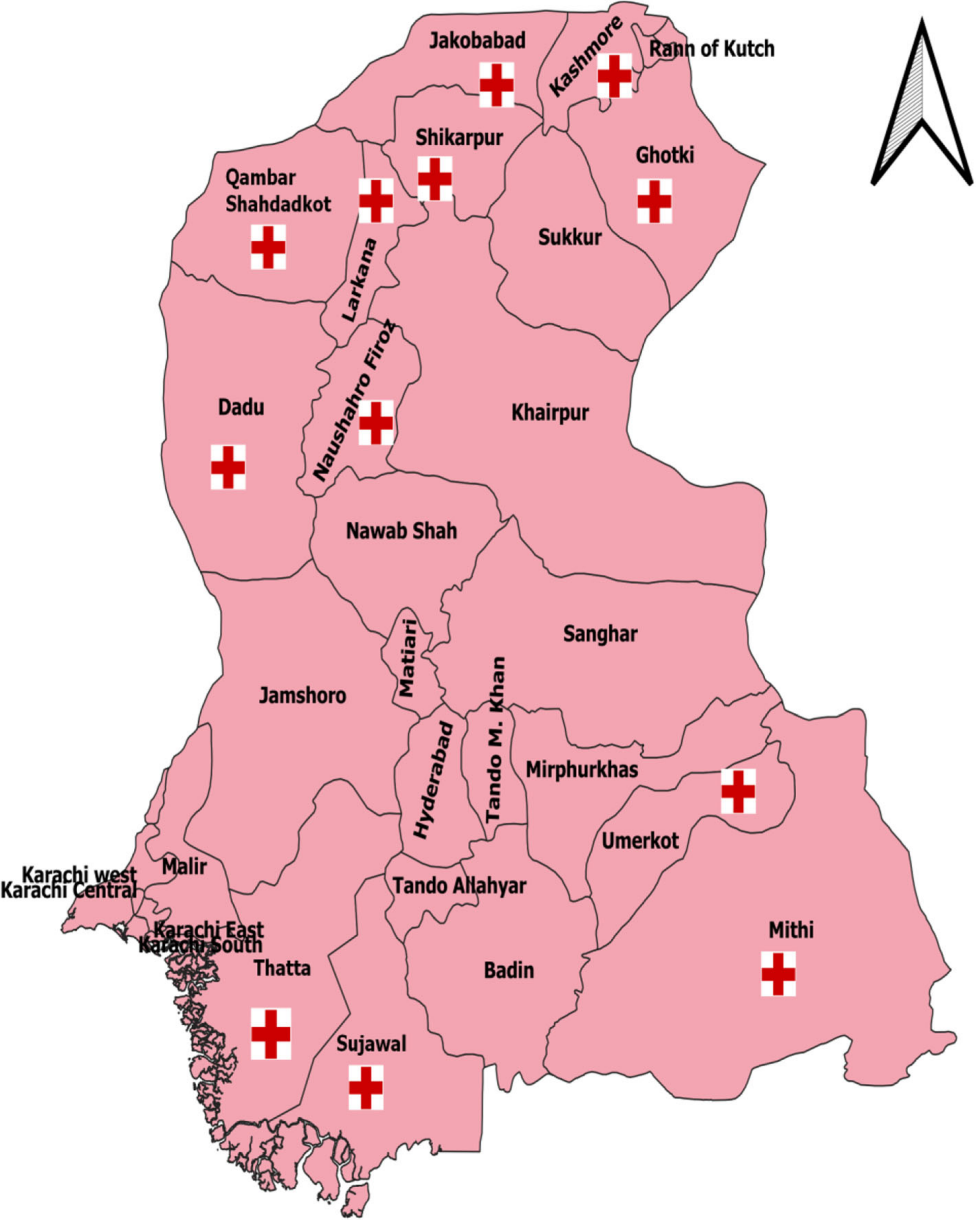
Geographical distribution of twelve districts surveyed in Sindh province (Developed by using WHO health mapper freely available; https://health-mapper.informer.com/4.3/)

This cross-sectional study was conducted in twelve of the 29 districts of Sindh Province (Fig. 1). The province had a total population of 47.9 million in 2017 [12]. These districts had poor maternal and child health indicators according to a ranking based on the multiple indicator cluster survey conducted in Sindh in 2014. This Survey was designed to provide estimates for more than 100 indicators about the women and children’s health and social status [13]. The main maternal and child healthcare indicators in the survey were: antenatal and postnatal care, contraception use rate, breastfeeding rate, vaccination coverage, institutional births with skilled providers and proportion of low birth weight babies. We adopted universal sampling technique by interviewing all hospital administrators from 25 RHC, 28 THQ, and 10 DHQ hospitals (Table 1). Questionnaire from World Health Organization (WHO) monitoring emergency obstetric care tool in developing countries was adapted and used after pretesting in adjacent district of Sindh and the availability of EmONC signal functions was assessed by adopting the direct observations WHO recommended check list during this study as described in Table 2 [14, 15]. The tool comprised of items on demographic information, availability of equipment and instruments necessary for EmONC. It also contained a checklist to assess EmONC and other maternal child healthcare services like; availability of newborn ward, kangaroo mother care, skilled birth attendance, caesarean section, facility environment and assessment of signal functions of BEmONC and CEmONC. A team of data collectors was trained and data collection quality was ensured by the principal investigator. The data were entered in Microsoft Excel and imported into Statistical Package for Social Sciences version 20 for analysis.

**Table 1:** Sociodemographic characteristics of the health facilities assessed in Sindh province (*n* = 63)

Selected Districts	Population		Number of district headquarters hospitals		Number of Taluka headquarters hospitals		Number of Rural Health Centers		Total number of health facilities visited
	Area (Km^2^)	Catchment population	Total	Selected	Total	Selected	Total	Selected	
Dadu	2551	1,089,169	1	1	4	3	5	1	5
Ghotki	19,808	1,649,661	1	1	5	3	3	1	5
Jacobabad	7705	979,817	1	1	3	2	3	3	6
Kamber Shahdadkot	2027	1,612,373	1	1	7	4	4	2	7
Kashmore	2771	1,006,297	1	–	3	1	4	2	3
Larkana	2577	1,231,481	1	–	4	3	5	2	5
Mithi/ Tharparkar	5599	1,341,042	1	1	6	2	2	1	4
Nausheroferoz	6506	1,647,239	1	1	5	2	12	2	5
Shikarpur	1906	1,524,391	1	1	4	2	9	4	7
Sujawal	8699	781,967	1	1	4	2	2	2	5
Thatta	8034	1,550,266	1	1	3	1	6	4	6
UmerKot	5503	1,073,146	1	1	5	3	6	1	5
Total			12	10	53	28	61	25	63

**Table 2:** Reported emergency obstetric and neonatal care signal functions in the health facilities of Sindh Province

BEmONC Signal functions	All health facilities (*n* = 63)		RHCs (*n* = 25)		THQs (*n* = 28)		DHQs (*n* = 10)	
	n(%)	95%CI	n(%)	95%CI	n(%)	95%CI	n(%)	95%CI
1. Parenteral antibiotics	58(92)	92(88.0–96.0)	23(92)	92(75.0–97.0)	25(89)	89(72.0–96.0)	10(100)	100(72.0–100.0)
2. Parenteral Oxytocin	57(90)	90(80.0–95.0)	22(88)	88(70.0–95.0)	25(89)	89(72.0–96.0)	10(100)	100(72.0–100.0)
3. Parenteral anticonvulsants	54(83)	83(75.0–92.0)	21(84)	84(65.0–93.0)	25(89)	89(72.0–96.0)	8(80)	80(49.0–94.0)
4. Manual removal of placenta	58(92)	92(88.0–96.0)	23(92)	92(75.0–97.0)	26(93)	92(77.0–98.0)	9(90)	90(59.0–98.0)
5. Removal of retained products	55(87)	87(76.0–93.0)	20(80)	80(60.0–91.0)	25(89)	89(72.0–96.0)	10(100)	100(72.0–100.0)
6. Assisted vaginal delivery	52(83)	82(71.0–89.0)	19(76)	76(56.0–88.0)	23(82)	82(64.0–92.0)	10 (100)	100 (72.0–100.0)
7. Neonatal resuscitation	51(81)	80(69.0–88.0)	20(80)	80(60.0–91.0)	22(79)	78(60.0–89.0)	9(90)	90 (59.0–98.0)
CEmONC Services								
1. Caesarean birth	15(24)	23(14.0–35.0)	0 (0)	0(0.0–13.0)	9(54)	32(17.0–50.0)	6(60)	60(31.0–83.0)
2. Blood Transfusion	36(57)	57(44.0–68.0)	11(44)	44(26.0–62.0)	16(57)	57(39.0–73.0)	9(90)	90(59.0–98.0)

Ethical approval was obtained from the Institutional Review Board of Health Services Academy, Islamabad; administrative permission from health department of Sindh province was also taken for the study.

## Results

### EmONC service availability in the health facilities

From the 63 health facilities surveyed, 25 (40%) were RHCs; and 28 (44%) and 10 (16%) were THQ and DHQ hospitals, respectively. Most of the health facilities 58(92%) surveyed had parenteral antibiotics available, 57(90%) of them had uterotonic drugs available for the management of the third stage of labor and postpartum hemorrhage prevention. Fifty two (83%) facilities had trained staff available to manage eclampsia and pre-eclampsia by anticonvulsant drug administration and 58(92%) had trained staff available to perform manual removal of placenta. Most 55(87%) health facility staff reported that they could perform for dilatation and curettage and vacuum extraction of retained products of placenta and 52(83%) facilities reported presence of services for assisted vaginal delivery. Newborn care services were available in 51(81%) of the health facilities. The availability of the BEmONC signal functions was better in DHQ hospitals compared to RHCs and THQs. However, with regard to CEmONC services, only 15(24%) of the health facilities reported offering caesarean section services and blood transfusion service was available only in 36(57%) of the health facilities (Table 2). These services have been verified through observation of the previous hospital record.

### Maternal and child healthcare service-related structures and services

Almost all facilities had antenatal care clinics except three health centers, including RHC New Jatoi, Buxapur and THQ hospital Kashmore. A postnatal ward was available at all DHQ and 18 THQ hospitals. A newborn ward was available in nine DHQ hospitals and about half of THQ hospitals. Labor rooms were available in all surveyed DHQ and THQ hospitals and RHCs. Equipment for assisted birth (vacuum extraction) was present in all DHQ and 26 THQ hospitals, and in 23 RHCs. Services for the active management of third stage of labor were available in most (84%) health facilities, and services for the management of pre-eclampsia and eclampsia were available in 81% of the health facilities and newborn resuscitation service was present in 85% of the health facilities. Post abortion care services, like manual vacuum aspiration and use of misoprostol were available in 81% of the health facilities. Protocol for assisted vaginal delivery was present in 84% facilities. However, a proper referral mechanism did not exist between these facilities (Table 3).

**Table 3:** Reported availability of maternal and child health related services in surveyed health facilities of Sindh Province

Indicators	All health facilities (*n* = 63)		RHCs (*n* = 25)		THQs (*n* = 28)		DHQs (*n* = 10)	
	*n*	Percent (95% CI)	*n*	Percent (95% CI)	*n*	Percent (95% CI)	*n*	Percent (95% CI)
Adequate number of beds	60	95(86.0–98.0)	24	96(80.0–99.0)	26	92(77.0–98.0)	10	100(72.0–100.0)
Newborn ward	37	57(46.0–70.0)	13	52 (33.0–69.0)	15	53(35.0–70.0)	9	90(59.0–98.0)
Kangaroo mother care area /room	29	45(34.0–58.0)	11	44(26.0–62.0)	12	42(26.0–60.0)	6	60(31.0–83.0)
Postnatal ward	4	71(60.0–82.0)	18	72(52.0–85.0)	18	64(45.0–79.0)	10	100(72.0–100.0)
Labor room	63	100 (94.0–100.0)	25	100(86.0–100.0)	28	100(87.0–100.0)	10	86(72.0–90.0)
Assisted birth (vacuum extraction)	59	93(84.0–97.0)	23	92(75.0–97.0)	26	89(77.0–98.0)	10	100(72.0–100.0)
Skilled birth attendants (nurse, midwife, Lady health visitor and/or a medical doctor)	61	95(89.0–99.0)	24	96(80.0–99.0)	27	96(82.0–99.0)	10	100(72.0–100.0)
Stock out of EmONC specific medicines is available as per Government supply	47	73 (62.0–83.0)	17	68 (48.0–82.0)	21	75(56.0–87.0)	9	90(59.0–98.0)
Facilities ready to provide EmONC services as per WHO criteria	46	71(60.0–82.0)	16	64(44.0–79.0)	22	80(60.0–89.0)	8	80(49.0–94.0)
Facilities with free caesarean-section	25	39(28.0–52.0)	16	64(44.0–79.0)	22	78(60.0–89.0)	8	80(49.0–94.0)

### Assessment of guidelines and policies for maternal and child healthcare services

Guidelines and polices related to maternal and child healthcare provision, case management and quality control mechanism were available in most of the surveyed health facilities; however, these guidelines were more likely to be available within DHQ hospitals compared to lower level health facilities. Less than half of the healthcare staff including doctors and paramedics had received any refresher training on EmONC service provision in the past 6 months. Most health facilities responded that they were gathering and communicating health data regularly (Table 4).

**Table 4:** Reported availability of guidelines and policies supporting maternal and child health Services in surveyed health facilities of Sindh province

EmONC service monitoring indicators	All health facilities (*n* = 63)		RHCs (*n* = 25)		THQs (*n* = 28)		DHQs (*n* = 10)	
	*n*	Percent (95% CI)	*n*	Percent (95% CI)	*n*	Percent (95% CI)	*n*	Percent (95% CI)
Availability of MNCH policy and guideline documents	49	77(66.0–86.0)	18	72(52.0–85.0)	21	75(56.0–87.0)	10	100(72.0–100.0)
EmONC quality check-up team nominated at the hospital	44	69(57.0–79.0)	18	0.72(52.0–85.0)	17	60(42.0–76.0)	9	90(59.0–98.0)
Regular data reporting	63	100(94.0–100.0)	25	100(86.0–100.0)	28	100(87.0–100.0)	10	100(72.0–100.0)
Facilities supervised in last 3 months	60	95(86.0–98.0)	23	92(75.0–97.0)	27	96(82.0–99.0)	10	100(72.0–100.0)
Any training in EmONC in 6 months	31	49(37.0–61.0)	10	40(23.0–59.0)	15	53(35.0–70.0)	6	60(31.0–83.0)
Availability of Soap/running water or alcohol rub in Labor Room	56	88(78.0–94.0)	20	80(60.0–91.0)	26	92(77.0–98.0)	10	100(72.0–100.0)
Any referral mechanism for patients during emergency	56	88(78.0–94.0)	21	84(65.0–93.0)	25	89(72.0–96.0)	10	100(72.0–100.0)

## Discussion

In the present study, availability of BEmONC and CEmNOC signal functions was assessed at the three levels of health care system in twelve districts of Sindh province where the status of maternal and child health, and other social indicators has been historically low. Even though most of the health facilities we surveyed reported high availability of the BEmONC services, the availability of CEmONC sginal functions of cesarean section and blood transfusion was substandard. Almost half of these health facilities lacked necessary guidelines and policies to manage maternal and child health related cases and about half of their staff lacked a recent training on maternal and child health related topic. Our findings are consistent with a previous multi-country survey which reported high availability of BEmONC services [16]. Studies have consistently showed that a low EmONC coverage in developing countries is linked with poor maternal and child health indicators [17–19]. The reason for this is that very few health facilities provide accessible caesarean section and blood transfusion services in many developing countries [15]. Our results are also consistent with other similar studies which show a reasonably acceptable availability of basic services; yet more advanced maternal and child health services have been lacking; thereby limiting communities’ access to these services and resulting in poor outcomes around birth [20–22]. Most of the maternal and newborn deaths in Pakistan occur at birth and improving access to EmONC services can tremendously improve birth outcomes [9].

Availability of CEmONC signal functions in secondary and tertiary level health facilities is necessary to ensure women and children have access to essential emergency care at birth. Not only the THQ hospitals in our study had low availability of caesarean section service; alarmingly, even most of the DHQ hospitals lacked it. Considering that these districts are geographically large, unavailability of an affordable caesarean section service within their main hospitals may result in access related challenges to the communities. These communities may not afford expenses related to caesarean birth in private hospitals and possibly experience hardships while travelling to the districts where such care may be available. This usually leads to families spending out of pocket leading to catastrophic expenditures. Nevertheless, private hospitals in some districts offer an alternative to absent caesarean section service in public sector hospitals [11].

As with caesarean section, blood transfusion availability as part of the national blood transfusion system, even in the hospitals offering CEmONC services, is still a challenge in countries like Pakistan. Previous research has also identified similar challenges to the provision of blood transfusion in other parts of Pakistan [21], as well as in other countries including Malawi and India which also have high maternal and child mortality [22]. Most patients in Pakistan access blood transfusion service through private blood transfusion centers by paying out of pocket; these facilities often require patients to arrange a blood donor [23]. Ensuring the availability of blood transfusion facilities could prevent a significant proportion of maternal deaths This is crucial considering that post-partum hemorrhage causes one-fourth maternal mortality burden in the developing countries [24].

Inadequately trained staff with less exposure to refresher trainings was another aspect of poor readiness of the health facilities to provide EmONC services. Availability of skilled and trained human resources for health could also be another challenge for the high maternal mortality ratio in surveyed districts, because employees are often poorly satisfied with their jobs and their work environment is usually suboptimal [25, 26]. Adequate training of health workers is another area of concern where the trained staff would be able to perform their responsibilities more efficiently which can impact better maternal and newborn health outcomes [27].

### Strengths and limitations

Direct access to the health facilities, use of a standard validated checklist and observations of the key structures and functions in the facilities was a strength of this study. However, since our data collectors relied upon health facility staff and managers for reporting, the actual availability of the services could be lower than is presented. Also, observations of the health facilities were conducted, yet they did not focus on the real time utilization of services by the patients. Although, it was found that the equipment and infrastructure for emergency obstetric care were available in the 12 surveyed districts of Sindh, and some of the health facilities also provided CEmONC services, the quality and patient satisfaction with the range of services was not assessed as it was not one of the aims of the present study.

## Conclusion

Although BEmONC service coverage was high in most of the health facilities in the province; the CEmONC service availability was dismal. Ensuring the availability of cesarean section and blood transfusion facilities in most strategically located secondary and tertiary care health facilities will improve the communities’ access to these two essential services and may lead to better maternal and newborn survival. Further research is recommended to directly determine the utilization and quality of EmONC services in the health facilities.

## Data Availability

The datasets used and/or analyzed during the current study are available from the corresponding author on reasonable request.

## Abbreviations

BEmONC: Basic Emergency Obstetric and Newborn care
CEmONC: Comprehensive Emergency Obstetric and Newborn care
EmONC: Emergency Obstetric and Newborn Care
